# Video analysis of potential concussions in elite male Hurling: are players being assessed according to league guidelines?

**DOI:** 10.1007/s11845-021-02798-7

**Published:** 2021-10-19

**Authors:** Darek Sokol-Randell, Mario Pasquale Rotundo, Gregory Tierney, Michael D. Cusimano, Conor Deasy

**Affiliations:** 1 School of Medicine, University College Cork, Cork, Ireland; 2 Emergency Innovation Research Network, Cork, Ireland; 3 School of Biomedical Sciences, University of Leeds, Leeds, UK; 4 Division of Neurosurgery, St. Michael’s Hospital, University of Toronto, Toronto, Canada; 5 Emergency Department, Cork University Hospital, Cork, Ireland

**Keywords:** Concussion assessment, Video incident analysis, Sport-related concussion

## Abstract

**Background:**

Hurling is a fast-paced contact sport that places players at risk of concussion. Given the consequences of repeated concussive impacts, it is imperative that concussion management guidelines are followed.

Hypothesis/Purpose.

The aim of this study is to determine if potential concussive events (PCEs) in elite Hurling are assessed in accordance with league management guidelines. The secondary objective is to investigate the effectiveness of current concussion training programs.

**Methods:**

Investigators used a video analysis approach to identify PCEs throughout the 2018 and 2019 inter-county Hurling seasons and championships. Subsequent assessment, return to play (RTP) decision, and signs of concussion were evaluated based on previously validated methods. The results were then compared year-over-year with previous research in Gaelic Football (GF).

**Results:**

A total of 183 PCEs were identified over 82 matches. PCEs were frequently assessed (86.3%, *n* = 158) by medical personnel. The majority of assessments were less than 1 min in duration (81.0%, *n* = 128). Thirteen (7.1%) players were removed following a PCE. There were 43 (23.5%) PCEs that resulted in one or more signs of concussion, of which 10 (23.3%) were removed from play. There was no difference in rate of assessment, duration of assessment, or rate of RTP between 2018 and 2019 in both Hurling and GF, suggesting that current concussion training programs have had limited success.

**Conclusion:**

In Hurling, players suspected of having sustained a concussion are frequently subject to a brief assessment, and are rarely removed from play. Affirmative action is needed to ensure the consistent application of standardized concussion assessment across the Gaelic Games.

## Introduction

In recent years, concussion has been identified as a major health concern in high-impact sports [1–3]. While the details and implications of this phenomenon are becoming increasingly well documented in the literature, there is little research addressing it in less globalized and amateur sports played around the world. The Gaelic Athletic Association (GAA) is the largest sporting organization in the Republic of Ireland. One of the most popular sports within their purview is Hurling, a fast-paced contact sport played on grass between two teams of 15 players, sometimes referred to as the fastest field sport on earth. Players are required to wear helmets, full metal faceguards, and mouthguards while using a wooden/composite hurling stick (hurley) to advance a small, hard leather ball (a sliotar) down the pitch. The sliotar can be caught in the hand and carried for up to four steps, struck in the air, or struck on the ground with the hurley. To carry the sliotar for more than four steps, the player must balance or repeatedly bounce the sliotar on their hurley. This requires remarkable hand–eye coordination at high-speeds, as players advance the sliotar down the pitch and bat it either between the uprights or into the goal. While outright tackling is not permitted as it is in sports such as rugby, shoulder-to-shoulder contact is encouraged and high-velocity collisions are commonplace [4, 5]. Players frequently execute tackles with their hurley as they attempt to block, hook, and swat the sliotar from their opponents.

In 2017, a survey of 80 GAA athletes revealed that 54% reported a personal history of concussion, with 44% reporting more than one incidence [6]. Confirming this, a 2019 survey suggested that while 57.5% of GAA athletes suspected they had suffered a concussion in the past, many were undiagnosed [7]. Our recent research in Gaelic Football, another national sport in Ireland that is also governed by the GAA, reported 2.2 potential concussive events (PCEs) per match at the elite inter-county level [8]. While this evidence suggests that concussion may occur relatively frequently in the Gaelic Games, diagnosed injuries to the head and neck have been shown to be relatively rare in Hurling, comprising of only 4.1% of all injuries [9]. It is possible that there is a mismatch between the number of concussions that are occurring and the number actually diagnosed. Although concussion awareness in the GAA has recently increased [10–12], there is little research investigating whether potential concussions are being appropriately identified and managed in Hurling.

In various sports, repeated concussive and sub-concussive blows to the head have been shown to produce a variety of neuro-cognitive and emotional deficits in players [13–15]. In the long-term, chronic progressive neurodegenerative diseases such as chronic traumatic encephalopathy (CTE) may affect athletes who suffer repeated blows to the head [16, 17]. In the acute phase, players who suffer a second head injury before a primary concussion has resolved are at risk of a severe condition called second-impact syndrome, marked by dysautoregulation, catastrophic cerebral swelling, and death [18, 19]. While this phenomenon has never been officially documented in the Gaelic Games, it is extremely dangerous, and documented cases in other sports are worth considering [19, 20]. It is important that players are made aware of these risks and feel reassured by the systems in place to protect them. While it is impossible to eliminate concussion from sports entirely, it is imperative that sporting leagues take measures to optimize the identification, assessment and substitution of athletes suspected of having sustained a concussion [21]. Previous peer-reviewed research has applied video-analysis of PCEs and subsequent management to investigate this topic [8, 22]. While a PCE may not necessarily result in clinical diagnosis, the definition encompasses a broad spectrum of head impacts that place players at risk of concussion [8, 22]. Therefore, PCEs may serve as a good proxy for investigating concussion management.

The 5th International Conference on Concussion in Sport (ICCS) recommended that all players suspected of having sustained a concussion should be removed from play and assessed using an accepted international standardized protocol, such as the Sports Concussion Assessment Tool 5 (SCAT-5). The GAA has integrated these recommendations independently through the Concussion Management Guidelines for Gaelic Games [10], and through a concussion training event held in November 2018 at Croke Park in Dublin, in conjunction with the UPMC Concussion Network [12]. However, little research exists on whether or not these measures have been effective in encouraging adherence to concussion management guidelines on the pitch. Recently, we investigated the assessment of PCEs in inter-county Gaelic Football, and concluded that there was limited adherence to league concussion guidelines over two seasons [8]. Despite the obvious differences between the two sports, the GAA concussion management guidelines are generalized and applicable to both Gaelic Football and Hurling. Thus, comparing PCE assessment data from both sports may provide a more accurate measure of adherence to these guidelines across the organization. Therefore, the current study will use video incident analysis to investigate PCEs that occurred during 2018 and 2019 elite inter-county Hurling. The primary objective is to determine if PCEs in Hurling are assessed in accordance with ICCS and GAA concussion guidelines. The findings will then be compared with previous findings in Gaelic Football to determine if there is any difference between the sports, and analyzed year-over-year to suggest whether current GAA concussion training programs have affected assessment practices across the organization.

## Methods

Video analysis has proven to be a valid method of analyzing situational factors, mechanisms, and signs of injury related to concussion [23–25]. Consistent with prior work, a PCE is defined as any event in which a player is unable to resume play in a meaningful capacity within 5 s of a direct and visible head contact [8, 22, 26]. In other words, the player was either down on the ground, staggering and/or bent over, therefore not participating purposefully or efficaciously in the gameplay around them. It is important to emphasize that the term PCE is not synonymous with concussion; the definition is intentionally broad to capture all events that have the potential to produce a concussion. Ambiguous events were excluded, such as those involving clear player embellishment, questionable head contact, or minor contact where the blow could not possibly produce a concussive force.

Reviewers were trained in video analysis at the Injury Prevention Lab at St Michael’s Hospital in Toronto, Ontario, Canada. The authors have previously published PCE research in professional soccer and Gaelic Football [8, 22, 26]. Match footage was retrieved from the GAAGO online streaming service and was analyzed using QuickTime Player v10.5 which enables frame-by-frame viewing at 720–1080p resolution. Reviewers were permitted to re-watch and pan the footage at their discretion. In player-to-player contact, we defined “Player 1” (P1) as the player who sustained head contact and “Player 2” (P2) as an involved player who did not. If both players sustained head contact during the PCE, P1 was defined as the one who was assessed for a longer period of time. Reviewers recorded whether the injured player was assessed by medical personnel, the duration of assessment and subsequent RTP decision. In light of the GAA “blood-sub” rule requiring that all players with visible bleeding be temporarily removed, reviewers also recorded whether or not each PCE produced blood. The time of the match at which each PCE occurred was also recorded to search for a relationship with the RTP decision. Finally, consistent with our prior work and a recent international consensus statement, reviewers searched for visible signs of concussion, including *lying motionless*, *impact seizure*, *tonic posturing*, *motor incoordination* — *ataxia*, *no protective action—floppy*, and *blank/vacant look* [18–20]. However, given the usage of helmets and faceguards, it sometimes proved challenging to evaluate for *blank/vacant look*. A PCE assessment spreadsheet, used in previous peer-reviewed research, was used to collect the data [8, 22]. Data from previous research in Gaelic Football [8] was also compared to data collected in the current study. Ethical approval was granted by the Social Research Ethics Committee of the Cork Teaching Hospitals (Ref: 10/09/2019/02).

To test for reliability, five non-2018/2019 matches were analyzed independently by both reviewers for raw agreement in the identification of PCEs. Second, each reviewer analyzed 30 PCEs identified from exhibition and All-Ireland club matches (not included in data analysis) using the PCE assessment spreadsheet. Agreement was 100.0% for the identification of PCEs. For the PCE assessment spreadsheet, raw agreement was 96.5% with a Cohen’s kappa coefficient of 0.93 (95% CI 0.884 to 0.969). A Cohen’s Kappa value greater than 0.8 is indicative of almost perfect agreement [21, 22]. All discrepancies from the inter-rater reliability were discussed and resolved.

In summary, after achieving a favorable inter-rater reliability result, two reviewers independently identified PCEs throughout 82 matches of the 2018 and 2019 GAA inter-county Hurling seasons and championships. Each PCE was evaluated based on the parameters outlined above, and data was recorded for statistical analysis. Reviewers consulted each other and collaborated if any questions or difficulties arose during the analysis. The results were compared to identical data collected in Gaelic Football [8], and stratified by year.

Descriptive statistics were reported as means, counts, or frequencies and their associated percentages. A Fisher’s exact test was used to confirm a relationship between the duration of assessment and multiple signs of concussion. In addition, Fisher’s exact test was used to search for an association between RTP decision and visible bleeding by varying number of concussion signs. A chi-squared test was used to analyze the two samples of independent variables from each year to determine if rate of assessment, duration of assessment, or rate of removal was significantly different between 2018 and 2019. Statistical significance was set at *p* < 0.05 for all statistical analyses.

## Results

Throughout the 2018 and 2019 GAA inter-county hurling seasons and championships, we identified a total of 176 incidents over 82 matches. Seven of these involved both P1 and P2 sustaining a PCE, resulting in 183 PCEs (2.2 per match, 59.5 per 1000 match hours of exposure) (Table 1). Of the 183 PCEs, 158 (86.3%) were assessed by a doctor and/or physiotherapist, identifiable by the lettering on their attire. The majority of these assessments were under 1 min in length (81.0%, *n* = 128). All assessment duration data is summarized in Table 1. Considering all on-pitch assessments, there was a significant association between the number of concussion signs and duration of assessment. Players displaying multiple signs of concussion were significantly more likely to receive a longer assessment (*p* < 0.05).

**Table 1:** Number of concussion signs by number of PCEs, assessment duration, player outcome, and visible bleeding (%) in GAA elite inter-county Hurling

Number of PCEs		Assessment duration							Player outcome					Bleeding
Number of concussion signs	2018–2019 Seasons	0–29 s	30–59 s	1:00–1:29	1:30–1.59	2:00 +	Removed	Total no. (%)	RTP with no assessment no. (%)	RTP after on-pitch assessment no. (%)	RTP after removal to SL no. (%)	Removed no. (%)	Total no. (%)	Bleeding no. (%)
0	140 (76.5)	65 (83.3)	38 (76.0)	9 (90.0)	0 (0.0)	0 (0.0)	6 (31.6)	**118 (74.7)**	22 (15.7)	112 (80.0)	3 (2.1)	3 (2.1)	**140 (76.5)**	4 (2.9)
1	31 (16.9)	13 (16.7)	10 (20.0)	0 (0.0)	0 (0.0)	0 (0.0)	5 (26.3)	**28 (17.7)**	3 (9.7)	23 (74.2)	3 (9.7)	2 (6.5)	**31 (16.9)**	2 (6.7)
2	6 (3.3)	0 (0.0)	1 (2.0)	1 (10.0)	1 (100.0)	0 (0.0)	3 (15.8)	**6 (3.8)**	0 (0.0)	3 (50.0)	0 (0.0)	3 (50.0)	**6 (3.3)**	1 (16.7)
3	3 (1.6)	0 (0.0)	0 (0.0)	0 (0.0)	0 (0.0)	0 (0.0)	3 (15.8)	**3 (1.9)**	0 (0.0)	0 (0.0)	0 (0.0)	3 (100.0)	**3 (1.6)**	0 (0.0)
4 +	3 (1.6)	0 (0.0)	1 (2.0)	0 (0.0)	0 (0.0)	0 (0.0)	2 (10.5)	**3 (1.9)**	0 (0.0)	1 (33.3)	0 (0.0)	2 (66.7)	**3 (1.6)**	0 (0.0)
Total	**183 (100.0)**	**78 (49.4)**	**50 (31.6)**	**10 (6.3)**	**1 (0.6)**	**0 (0.0)**	**19 (12.0)**	**158 (100.0)**	**25 (13.7)**	**139 (76.0)**	**6 (3.3)**	**13 (7.1)**	**183 (100.0)**	**7 (3.8)**

We observed 140 (76.5%) PCEs that displayed 0 signs of concussion and 43 (23.5%) that displayed 1 or more. Of the 43 PCEs that produced 1 or more sign of concussion, 10 (23.3%) were removed from play. This data is summarized in Table 1. Players displaying multiple signs of concussion were significantly more likely to be removed from play than players with 0 signs (*p* < 0.01).

Twenty-five (13.7%) players suffering a PCE received no assessment before RTP, while 139 (76.0%) players were assessed on pitch before RTP. Six (3.3%) players were taken to the sideline before RTP (Table 1),three of these six players (50.0%) were visibly bleeding following the associated PCE (Table 2), and 1 (16.7%) was treated for a hand injury. Thirteen (7.1%) players were removed from play and did not RTP for the rest of the game (Table 1), two of these 13 (15.4%) players were visibly bleeding (Table 2), and 1 (7.7%) suffered the associated PCE in the final 5 min of the match. Overall, 7 (3.8%) PCEs produced visible signs of bleeding, and 5 (71.4%) of these players were removed either temporarily or permanently (Table 2). Players were significantly more likely to be removed if bleeding was present (*p* < 0.05).

**Table 2:** RTP decision and bleeding (%)

RTP decision	No. of players	Bleeding
RTP with no assessment	25	0 (0.0)
RTP after on-pitch assessment	138	2 (28.6)
RTP after removal to SL	6	3 (42.9)
Removed	13	2 (28.6)
Total	**182**	**7 (3.8)**

Our findings in Hurling were compared to previous research performed in Gaelic Football and stratified by year (Table 3). In both Gaelic Football and Hurling, there was no difference in the rate of assessment, duration of assessment, or rate of player removal between 2018 and 2019 (*p* > 0.1) (Table 3).

**Table 3:** Major assessment and RTP findings in GAA elite inter-county Hurling and Gaelic Football, over the 2018 and 2019 seasons

Category	Hurling			Gaelic Football		
*YEAR*	*2018*	*2019*	*TOTAL*	*2018*	*2019*	*TOTAL*
*Number of PCEs*	75	108	183	79	163	242
*PCE incidence (per 1000 match hours)*	48.8	70.2	59.5	35.71	83.59	58.1
*PCEs assessed (%)*	66 (88.0)	92 (85.2)	158 (86.3)	71 (89.9)	140 (85.9)	211 (87.2)
*PCEs not assessed (%)*	9 (12.0)	16 (14.8)	25 (13.7)	8 (10.1)	23 (14.1)	31 (12.8)
*Assessments* < *1 m in duration (%)*	54 (81.8)	74 (80.4)	128 (81.0)	57 (80.3)	116 (82.9)	173 (82.0)
*Assessments* > *1 m in duration (%)*	12 (18.2)	18 (19.6)	30 (19.0)	14 (19.7)	24 (17.1)	38 (18.0)
*RTP after assessment on pitch (%)*	57 (76.0)	82 (75.9)	139 (76.0)	61 (77.2)	128 (78.5)	189 (78.1)
*RTP after assessment on SL/off-pitch (%)*	2 (2.7)	4 (3.7)	6 (3.3)	7 (8.9)	3 (1.8)	10 (4.1)
*Removed from play (%)*	7 (9.3)	6 (5.6)	13 (7.1)	3 (3.8)	9 (5.5)	12 (5.0)
*1* + *Signs of Concussion (%)*	20 (26.7)	23 (30.7)	43 (23.5)	20 (25.3)	41 (25.2)	61 (25.2)
*Players with 1* + *signs of concussion, removed from play (%)*	6 (30.0)	4 (17.4)	10 (23.3)	3 (15.0)	6 (14.6)	9 (14.8)
*% adherence to blood-sub rule*	100.0	50.0	71.4	100.0	90.0	92.9

## Discussion

In GAA elite inter-county Hurling, players suffering a PCE were frequently assessed (86.3%). However, most of these assessments were under 1 min in duration (81.0%, *n* = 128). The GAA’s Concussion Management Guidelines recommend that athletes suspected of having sustained a concussion be removed from play immediately and medically assessed using the SCAT-5 protocol, which takes a *minimum* of 10 min to complete [27]. Unfortunately, throughout the 2018 and 2019 Hurling seasons, we were unable to identify any assessments that resembled the SCAT-5. Players are not receiving standardized assessment following PCEs and RTP at a high rate, even when visible signs of concussion are present. Those returning to play without being properly screened for concussion are at risk of further strikes to the head and more severe post-concussive symptoms and complications [14, 18, 28, 29]. Despite the wishes of the players, manager, or fans, medical staff must err on the side of caution when deciding whether to allow a player to RTP. The use of a standardized assessment protocol must be enforced to ensure that concussions are not missed, and that players only RTP if it is safe to do so.

We have previously discussed various barriers that medical personnel face with regard to PCE assessment in Gaelic Football, [8] and many of the same points also apply to Hurling. However, the current study has elucidated a number of additional challenges that medical personnel may face. First, since 2010, all Hurling players have been required to wear a helmet with faceguard, which may contribute to both the incidence and assessment of PCE. This legislation was intended to reduce the number of serious facial injuries in the sport, and research has demonstrated that this measure has been successful [34, 35]. Our data demonstrated that a lower proportion of Hurling players demonstrated bleeding following a PCE compared to Gaelic Football (Table 3), and players with multiple signs of concussion were not significantly more likely to bleed as they were in Gaelic Football [8]. This favorable effect could be related to the use of helmets and faceguards. However, while helmets and other forms of protective equipment have long been theorized to be protective against concussion, this may not necessarily be the case [36–38]. In rugby, it was discovered that the use of headgear has no effect on concussion risk [39]. In fact, there is evidence to suggest that headgear may increase concussion risk via a phenomenon known as *risk compensation*, which postulates that helmets confer their wearer with a false sense of security [40]. If a player perceives that his risk of injury is reduced by using a helmet, he may be more likely to take excessive and unwarranted risks during gameplay [40]. A survey of young rugby players suggested that 67.0% of players felt more confident when wearing headgear, and were able to tackle harder than they could without it [41]. This is especially relevant given that the helmets used in Hurling are not designed to defend against concussive impacts, as modern American football helmets are [42]. It is possible that this perceived reduction in risk extends to sideline medical staff, increasing their threshold for delivering a full assessment and ordering removal from play. If doctors and physiotherapists hold an implicit bias that helmets protect players from concussion, they may assume that players are not concussed and forego a full assessment when confronted with clinical ambiguity. In addition, the use of helmets may hinder the ability of sideline medical staff to both identify and assess PCEs, as they may disguise the nature and severity of an impact, as well as transient signs of concussion which are already notoriously difficult to see [21]. Our data supported this in that there was less adherence to the blood-sub rule in Hurling (71.4%) when compared to Gaelic Football (92.9%), possibly due to the disguising effect of the helmet. This reinforces the need for comprehensive, mandatory training programs for medical staff to develop an awareness of biases such as *risk compensation* and the effect of helmets on concussion risk [43]. It also underscores the importance of removing players suspected of having sustained a concussion, so they may remove their helmet and be thoroughly assessed.

Second, it is important to consider the needs of the medical professionals. Medical roles on GAA club teams are typically filled on a volunteer basis, meaning that many team physicians and physiotherapists generously donate their personal time to look after athletes. The implications of this must be investigated in more detail, especially with regard to time commitment, travel requirements, financial compensation, and burnout [30–33], all of which might impact a practitioner’s ability to administer high-quality care. Furthermore, concussion management is a fledgling field. Many professionals, despite years of experience, may not be fully up to date on recent developments in knowledge and protocol. It may be prudent for the league to provide increased support to its medical staff to ensure their ability to consistently and effectively perform their duties at training sessions and matches.

In recent years, the GAA has attempted to increase concussion awareness among its medical staff. Two concussion symposiums were hosted by the league in 2016 and 2017, and in November 2018, the GAA held its first concussion training event in conjunction with the UPMC Concussion Network. This program, designed for doctors and physiotherapists at the elite level, provided information and workshops on concussion diagnosis and assessment, as well as treatment and rehabilitation [12], based on the GAA’s universal set of concussion management guidelines. Given that the league presides over both sports, it was unsurprising to find that in both Gaelic Football and Hurling, assessment practices were relatively similar (Table 3). Unfortunately, our research suggests that the aforementioned concussion training events have had a limited effect on assessment practices. There was no significant difference in the rate and duration of assessment, or rate of player removal in 2019 compared to 2018 (Table 3). In both Gaelic Football and Hurling, players continue to RTP without adequate standardized assessment at an unacceptably high rate, often despite the presence of visible signs of concussion [8]. While the GAA commendably intends to “… grow a national network of support for the management and treatment of concussion injuries within the GAA” [12], our findings suggests that action must be taken to accelerate this process.

The organizational structure of the GAA confers a great advantage in that education and training programs for medical personnel can be standardized and disseminated across multiple sports from a centralized body. While the GAA’s existing training events are predominantly directed towards the elite level of Gaelic Football and Hurling, there are hundreds of other teams at all age groups across the country to consider. Although resources and manpower may be understandably limited across different levels of play, perhaps there is a practical way to overcome this challenge. A recent survey found that over 90% of adolescent GAA players and parents would value further education on sport-related concussion [7]. We recommend that educational training programs for players, coaches, volunteers, and parents are promoted and made available at all levels of play. The 2018 GAA concussion training event was based on a “train-the-trainer” model; perhaps, this can be applied at the club level as well. Representatives from each club who attend the GAA’s official annual training sessions might be tasked with delivering educational workshops locally. We would encourage leaders to take advantage of the revolution in remote educational strategies that have emerged during the COVID-19 pandemic, which facilitate group education across long distances. This can be delivered in conjunction with existing research on concussion education programs for adolescent GAA athletes [44], and would promote a grass-roots change in the way concussions are perceived and managed in the Gaelic Games.

Finally, a league-wide quality assurance program at the elite level should occur throughout each season to ensure that an appropriate level of concussion care is being offered to players. This can be achieved through a video surveillance operation using a protocol similar to the current study. In the same way, medical staff in healthcare systems are responsible for delivering a certain standard of care; staff in sports leagues such as the GAA must also be held to a standard. This is especially relevant at the elite level, which is highly publicized and sets the standard for other levels of play to emulate. Increased oversight from the GAA is necessary to ensure that its training events are producing the intended effects, and that its concussion management guidelines are actually reaching players on the pitch.

### Limitations

This study relied upon broadcaster game replays. Thus, reviewers were unable to control camera views, angles, and quality, impacting their ability to evaluate PCEs. It was also more difficult to note the presence of the *blank/vacant look*, given the visual obstruction of a helmet and faceguard. The statistics of PCE incidence and signs of concussion presented in this study likely represent a minimum estimate; the true incidence of PCE with transient signs of concussion may be higher. Additionally, reviewers had no access to in-game audio information to integrate into the analysis. It is also possible that players occasionally exaggerate or feign injury to gain ball possession or a freekick/penalty for their team. We made every effort to exclude these events. We had no access to medical reports from the games so we cannot infer which PCEs were actually associated with medically diagnosed concussion. Future research will help to elucidate this relationship.

Little is known about the reliability and validity of reviewers observing concussion signs on video analysis. Although the specific signs have been well defined through international consensus, it is difficult to infer the sensitivity of our analysis as there is no accepted “gold standard” identification system. Thus, there was inevitably an element of subjectivity in our analysis. Finally, as with any video review study involving more than one reviewer, we acknowledge the possibility that inter-reviewer bias may have played a role in data collection and analysis. However, we attempted to mitigate this by achieving both a high raw agreement and Cohen’s kappa coefficient.

## Conclusion

Our study demonstrates that elite GAA Hurling players frequently receive a brief assessment and almost always RTP following PCE. Assessment practices in Hurling did not appear to comply with GAA concussion guidelines, consistent with prior research in Gaelic Football. It is important that the GAA as an organization continues its efforts to standardize concussion assessment and RTP protocol across the Gaelic Games, at all levels of play. Medical staff providing cover for matches must be sufficiently supported and empowered to provide effective in-game concussion management. Ongoing training, education, and quality assurance programs are required to ensure coaches, referees, players, and parents appreciate the importance of concussion management, thereby supporting a more informed safety culture when it comes to head injury. We hope that increased collaboration amongst medical staff and the community will facilitate a collective improvement in concussion care for years to come.

## Data Availability

Data available upon reasonable request.

## References

[1] Meeuwisse WH, Schneider KJ, Dvořák J et al (2017) The Berlin 2016 process: a summary of methodology for the 5th International Consensus Conference on Concussion in Sport. Br J Sports Med 51:873–6. 10.1136/bjsports-2017-097569 10.1136/bjsports-2017-097569 28446449

[2] Koh JO, Cassidy JD, Watkinson EJ (2003). Incidence of concussion in contact sports: a systematic review of the evidence. Brain Inj.

[3] Cusimano MD, Cho N, Amin K (2013). Mechanisms of team-sport-related brain injuries in children 5 to 19 years old: opportunities for prevention. PLoS ONE.

[4] Ly N (2015) The Rules of Hurling - EXPLAINED! https://www.youtube.com/watch?v=biFcgUB98ns

[5] Kavanagh B (2015) GAA Biggest Hits | Hurling & Football |

[6] Sullivan L, Thomas AA, Molcho M (2017). An evaluation of Gaelic Athletic Association (GAA) athletes’ self-reported practice of playing while concussed, knowledge about and attitudes towards sports-related concussion. Int J Adolesc Med Health.

[7] O’Connor S, Moran K, Burke C, Whyte E (2019). Sports-related concussion in adolescent Gaelic Games players. Sports Health.

[8] Sokol-Randell D, Rotundo MP, Tierney G et al (2020) Frequent but limited assessment of potentially concussed players in Gaelic Football: an opportunity to learn from other sports. Irish J Med Sci (1971 -). 10.1007/s11845-020-02390-5 10.1007/s11845-020-02390-5 32997230

[9] Blake C, O’Malley E, Gissane C, Murphy JC (2014). Epidemiology of injuries in hurling: a prospective study 2007–2011. BMJ Open.

[10] Gaelic Athletic Association (2018) Concussion management guidelines for Gaelic Games

[11] UPMC Concussion Network

[12] Gaelic Athletic Association concussion care cultivated at Croke park with GAA & UPMC Concussion Network. https://www.gaa.ie/news/concussion-care-cultivated-at-croke-park-with-gaa-upmc-concussion-network/

[13] Stewart WF, Kim N, Ifrah C et al (2018) Heading frequency is more strongly related to cognitive performance than unintentional head impacts in amateur soccer players Front Neurol 9 10.3389/fneur.2018.00240 10.3389/fneur.2018.00240 PMC5928847 29740384

[14] Guskiewicz KM, Marshall SW, Bailes J (2007). Recurrent concussion and risk of depression in retired professional football players. Med Sci Sports Exerc.

[15] Killam C, Cautin RL, Santucci AC (2005). Assessing the enduring residual neuropsychological effects of head trauma in college athletes who participate in contact sports. Arch Clin Neuropsychol.

[16] Omalu B (2014). Chronic traumatic encephalopathy. Prog Neurol Surg.

[17] Mackay DF, Russell ER, Stewart K et al (2019) Neurodegenerative disease mortality among former professional soccer players N Engl J Med 1801–8 10.1056/NEJMoa1908483 10.1056/NEJMoa1908483 PMC8747032 31633894

[18] Cantu RC (2016). Dysautoregulation/second-impact syndrome with recurrent athletic head injury. World Neurosurg.

[19] McLendon LA, Kralik SF, Grayson PA, Golomb MR (2016) The controversial second impact syndrome: a review of the literature. Pediatr. Neurol. 10.1016/j.pediatrneurol.2016.03.009 27421756

[20] Tator C, Starkes J, Dolansky G (2019). Fatal second impact syndrome in Rowan Stringer, A 17-Year-Old Rugby Player. Can J Neurol Sci.

[21] McCrory P, Meeuwisse W, Dvořák J (2017). Consensus statement on concussion in sport-the 5th international conference on concussion in sport held in Berlin, October 2016. Br J Sports Med.

[22] Tarzi C, Aubrey J, Rotundo M (2020). Professional assessment of potential concussions in elite football tournaments. Inj Prev.

[23] Davis GA, Makdissi M, Bloomfield P (2018). International study of video review of concussion in professional sports. Br J Sports Med.

[24] Davis G, Makdissi M (2016). Use of video to facilitate sideline concussion diagnosis and management decision-making. J Sci Med Sport.

[25] Gardner AJ, Iverson GL, Stanwell P (2016). A video analysis of use of the new “concussion interchange rule” in the national rugby league. Int J Sports Med.

[26] Armstrong N, Rotundo M, Aubrey J (2019). Characteristics of potential concussive events in three elite football tournaments. Inj Prev.

[27] Echemendia RJ, Meeuwisse W, McCrory P et al (2017) The Sport Concussion Assessment Tool 5th Edition (SCAT5). Br J Sports Med bjsports-2017–097506. 10.1136/bjsports-2017-097506 10.1136/bjsports-2017-097506 28446453

[28] Asken BM, Bauer RM, Guskiewicz KM (2018). Immediate removal from activity after sport-related concussion is associated with shorter clinical recovery and less severe symptoms in collegiate student-athletes. Am J Sports Med.

[29] Asken BM, McCrea MA, Clugston JR (2016). Playing through it: delayed reporting and removal from athletic activity after concussion predicts prolonged recovery. J Athl Train.

[30] Gieck J, Brown R, Shank R (1982). The burnout syndrome among athletic trainers. Athl Train J Natl Athl Train Assoc.

[31] Dale J, Weinberg R (1990). Burnout in sport: a review and critique. J Appl Sport Psychol.

[32] McLaine AJ (2005) An overview of burnout in athletic trainers. Athl. Ther. Today

[33] Lemak L (2007) Financial implications of serving as team physician. Clin. Sports Med. 10.1016/j.csm.2007.01.003 17499625

[34] Khan MI, Flynn T, O’Connell E (2008). The impact of new regulations on the incidence and severity of ocular injury sustained in hurling. Eye.

[35] Murphy C, Ahmed I, Mullarkey C, Kearns G (2010) Maxillofacial and dental injuries sustained in hurling. Ir Med J 20669600

[36] Sone JY, Kondziolka D, Huang JH, Samadani U (2017) Helmet efficacy against concussion and traumatic brain injury: a review. J. Neurosurg. 10.3171/2016.2.JNS151972 27231972

[37] Daneshvar DH, Baugh CM, Nowinski CJ et al (2011) Helmets and mouth guards: the role of personal equipment in preventing sport-related concussions. Clin. Sports Med. 10.1016/j.csm.2010.09.006 PMC2987604 21074089

[38] Benson BW, Hamilton GM, Meeuwisse WH et al (2009) Is protective equipment useful in preventing concussion? A systematic review of the literature. In: Br. J. Sports Med. 10.1136/bjsm.2009.058271 19433427

[39] McIntosh AS, McCrory P, Finch CF (2009). Does padded headgear prevent head injury in rugby union football?. Med Sci Sports Exerc.

[40] Hagel B, Meeuwisse W (2004) Risk compensation: a “side effect” of sport injury prevention? Clin. J. Sport Med. 10.1097/00042752-200407000-00001 15273524

[41] Finch CF, McIntosh AS, McCrory P (2001). What do under 15 year old schoolboy rugby union players think about protective headgear?. Br J Sports Med.

[42] Post A, Oeur A, Hoshizaki B, Gilchrist MD (2013). An examination of American football helmets using brain deformation metrics associated with concussion. Mater Des.

[43] Herring SA, Cantu RC, Guskiewicz KM, Putukian M, Kibler WB, Bergfeld JA, American College of Sports Medicine (2011) Concussion (Mild Traumatic Brain Injury) and the team physician: a consensus statement-2011 update. Med. Sci. Sports Exerc. 10.1249/MSS.0b013e3182342e64 22089299

[44] Sullivan L (2018) The development, implementation, and evaluation of a theory-based concussion education programme for secondary school Gaelic Athletic Association (GAA) athletes. National University of Ireland Galway

